# Ultrasound-measured renal length and corticomedullary thickness for the diagnosis and staging of chronic kidney disease in cats

**DOI:** 10.1093/jvimsj/aalag274

**Published:** 2026-11-10

**Authors:** Marleen Brans, Emmelie Stock, Luc Duchateau, Dominique Paepe

**Affiliations:** Small Animal Department, Faculty of Veterinary Medicine, Ghent University, Merelbeke, Belgium; Department of Morphology, Imaging, Orthopedics, Rehabilitation and Nutrition, Faculty of Veterinary Medicine, Ghent University, Merelbeke, Belgium; Department of Morphology, Imaging, Orthopedics, Rehabilitation and Nutrition, Faculty of Veterinary Medicine, Ghent University, Merelbeke, Belgium; Small Animal Department, Faculty of Veterinary Medicine, Ghent University, Merelbeke, Belgium

**Keywords:** corticomedullary ratio, hyperthyroidism, observer variability, renal cortical thickness, renal medullary thickness

## Abstract

**Background:**

Ultrasonography is well established in veterinary practice, and renal dimensions may aid in diagnosing chronic kidney disease (CKD) in cats.

**Hypothesis/Objectives:**

Assess whether renal length, cortical and medullary thickness, and derived ratios identify CKD, reflect CKD severity, and are changed by hyperthyroidism. Evaluate effects of age, body weight, sex, laterality, and observer variability on renal dimensions.

**Animals:**

Two hundred fifty-nine client-owned cats: healthy (*n* = 179), CKD-affected (*n* = 38), and nonazotemic hyperthyroid (*n* = 42).

**Methods:**

Ultrasonographic renal dimensions were measured retrospectively. Group comparisons were performed using fixed effects and mixed models. Diagnostic accuracy was assessed using receiver operating characteristic analysis. Effects of age, body weight, sex, and laterality were evaluated using multiple linear regression. Intraobserver (novice) and interobserver (novice vs expert) variability were quantified using intraclass correlation coefficients from random effect models.

**Results:**

Age, body weight, and sex accounted for biological variation in healthy cats, which also exhibited minor left–right kidney asymmetry. Cats with CKD were reliably distinguished from healthy and hyperthyroid cats by renal dimensions. Compared with age-comparable healthy cats, CKD-affected cats had shorter kidneys (−0.49 cm, −13.26%, *P* < .001), thinner cortices (dorsal: −0.10 cm, −16.23%, *P* < .001; transverse: −0.06 cm, −9.14%, *P* < .05), and thinner medullae (dorsal: −0.28 cm, −26.25%, *P* < .001; transverse: −0.24 cm, −19.7%, *P* < .001). Renal length and medullary thickness showed the highest diagnostic accuracy, but no renal dimension reliably reflected disease severity. Interobserver variability was considerable.

**Conclusions and clinical importance:**

Ultrasonographic renal dimensions may support CKD diagnosis in cats, but biological and observer variability must be considered.

## Introduction

B-mode ultrasonography plays an important role in the diagnostic evaluation of cats with chronic kidney disease (CKD), identifying specific causes and morphological changes associated with impaired renal function.^1–3^

In human medicine, renal corticomedullary (CM) thickness has been shown to correlate with renal function.^3–5^ Decreased renal cortical thickness (RCT) was associated with decreased renal function, particularly in early CKD, whereas decreased renal medullary thickness (RMT) appeared mainly linked to advanced disease stages.^3^ Renal cortical thickness has been reported to correlate more closely with renal function than length, suggesting superior value as a marker of disease severity.^3–5^

In cats, one study identified a moderate to strong association between the RCT and renal function, with RCT outperforming RMT and renal length for CKD identification.^2^ Although RCT was not reliable for staging disease severity, it showed good reproducibility. However, this study included relatively few healthy controls.^2^

Beyond renal function, ultrasound-based renal dimensions may also be influenced by demographic and individual variables. In healthy cats, renal length has been shown to correlate significantly with body weight,^6–8^ sex,^2^^,^^6^^,^^7^ and age,^2^^,^^7^ but not with gonadal status.^6^^,^^9^ In contrast, RCT, RMT, and the CM ratio generally were unaffected by body weight,^2^^,^^7^ or age.^2^^,^^7^ Although some studies reported no effect of sex on RCT, RMT, or the CM ratio,^2^^,^^7^ others found a positive effect of male sex on CM thickness as well as a significant effect of gonadal status on RMT.^6^ Furthermore, both renal length^6^^,^^9^ and CM ratio^9^ were influenced by kidney laterality, with the left kidney being slightly smaller, exhibiting greater RMT, and consequently a lower CM ratio.^9^

Renal CM thickness in cats has been investigated in only a few studies, often involving small sample sizes and variable scan planes. It also remains unknown whether measurements are affected by hyperthyroidism, a common condition in senior cats that may mask early CKD.

In our retrospective study including a large sample of healthy and diseased cats, the primary objectives were: (1) to examine if ultrasound-measured renal dimensions (ie, renal length, CM thickness, and CM ratios in dorsal and transverse planes) can detect CKD; (2) to examine their association with disease severity; and (3) to evaluate the influence of hyperthyroidism on these variables. A secondary objective was to assess the extent to which renal dimensions vary with age, body weight, sex, and kidney laterality. Finally, inter- and intraobserver variability of the measurements was evaluated.

## Materials and methods

Our retrospective, observational study reviewed medical records of client-owned cats that underwent abdominal ultrasonography at the small animal clinic, Faculty of Veterinary Medicine, Ghent University. Cats were included if longitudinal, dorsal, and transverse sonograms of the kidneys were available and if the cats were healthy, or diagnosed with nonazotemic hyperthyroidism or CKD at the time of imaging. Health assessment included a medical history, physical examination, comprehensive blood testing and urinalysis, systolic blood pressure measurement, and abdominal ultrasonography. Cats were considered healthy if no clinically relevant abnormalities were present. They were subdivided by life stage.^10^ Azotemic CKD was diagnosed based on clinical signs with persistent renal azotemia. Nonazotemic CKD was defined by normal serum creatinine (sCr) concentration with other evidence of renal abnomalities.^11^ Hyperthyroidism was diagnosed by increased serum total thyroxine concentration and increased pertechnetate uptake on a scan performed using standard methodologies.^12^

The primary outcomes were ultrasound-based renal dimensions (renal length, RCT, RMT, and derived CM ratios). Ultrasonography was performed by a Diplomate of the European College of Veterinary Diagnostic Imaging (ECVDI) or supervised resident following a standardized protocol using a linear transducer (12-5 MHz) on dedicated machines.^13^ Two observers evaluated renal sonograms: an ECVDI diplomate (expert) and small animal veterinarian with minimal experience (novice). The expert measured all kidneys once; the novice measured one randomly selected kidney twice per cat. Interobserver variability was evaluated by comparing measurements of the same kidneys by both observers; intraobserver variability was assessed from the second observer’s replicate measurements. Observers were blinded to cat identity, clinical information, and health status.

A fixed effects model was used to compare renal dimensions across disease groups using a limited number of prespecified, biologically motivated pairwise contrasts, with transverse RCT defined a priori as the primary endpoint based on earlier reseach.^2^ Post-hoc comparisons within the CKD group across International Renal Interest Society (IRIS) stages were done using Tukey-adjusted *P*-values. Receiver operating characteristic (ROC) curves were constructed to assess the diagnostic capacity of renal dimensions in differentiating the CKD group from the senior healthy group. A mixed model with cat as random effect was applied to evaluate kidney laterality. Multiple linear regression was used to evaluate the effects of age, weight, and sex on renal dimensions in healthy cats. Inter- and intraobserver agreement were quantified using intraclass correlation coefficients (ICCs) from a random effects model. Bland–Altman (BA) plots were added to visualize inter- and intraobserver measurement differences and 95% limits of agreement (95% LoA).

Detailed materials, measurement protocols, and statistical procedures are provided in the Appendix.

## Results

### Study sample and descriptive statistics

Two hundred fifty-nine cats (European Shorthair *n* = 193, Ragdoll *n* = 21, British Shorthair *n* = 17, Persian *n* = 6, Maine Coon *n* = 4, European Longhair *n* = 3, Sphynx *n* = 3, Burmese *n* = 2, Eastern Shorthair *n* = 2, Angora *n* = 1, Bengal *n* = 1, Burmese *n* = 2, Chartreux *n* = 1, Norwegian Forest cat *n* = 1, Birman cats *n* = 1, Scottish Fold *n* = 1, Scottish Straight *n* = 1, and Siamese *n* = 1) were enrolled in the study and assigned to one of the following groups, defined by health status and secondarily by age for healthy cats: junior healthy group *n* = 45, young adult healthy group *n* = 45, mature adult healthy group *n* = 43, senior healthy group *n* = 46, CKD group *n* = 38, and hyperthyroid group *n* = 42. Cats with CKD were subdivided according to IRIS stage after diagnosis (stage 1 *n* = 3, stage 2 *n* = 21, stage 3 *n* = 13, and stage 4 *n* = 1).

Table 1 represents the main demographic characteristics and the routine renal biomarkers of the study sample categorized into (health × age) groups. In some cats, accurate measurements of one or more renal dimensions as described in the “Materials and method” section were not possible because of either missing images or insufficient image resolution (Table S1). Ultrasonographic renal abnormalities were commonly observed in the study sample. Irregular renal shape, solid nodules, dystrophic mineralization, nephrolithiasis, segmental cortical lesions, decreased CM distinction, and hyperechogenicity of cortex and medulla were more prevalent in CKD-affected cats. The medullary rim and band sign were present across all disease-age groups. The prevalence and distribution of all abnormalities are summarized in Table S2. Representative sonograms of kidneys from cats with and without renal abnormalities are shown in Figure 1.

**Table 1:** Summary of demographic variables: age (*n* = 259), body weight (*n* = 225), and sex (*n* = 259) and renal biomarkers serum creatinine (*n* = 257) and urine specific gravity (*n* = 252) of the complete study sample of cats classified according to health status. The healthy cats were subdivided into life stages based on age. Age, body weight, sCr, and USG are expressed as median and range. For sex and gonadal status, the exact numbers are given.

		**Junior (1-2 years)**	**Adult (3-6 years)**	**Mature adult (7-10 years)**	**Senior (>11 years)**	**CKD**	**Hyperthyroid**
		***n* = 45**	***n* = 45**	***n* = 43**	***n* = 46**	***n* = 38**	***n* = 42**
**Age in years**		1.67 (1.00-2.92)	4.83 (3.00-6.92)	9.42 (7.08-10.83)	12.17 (11.00-15.92)	12.13 (1.00-18.25)	12.75 (6.75-16.58)
**Body weight in kg^a^**		4.30 (2.60-5.85)	4.40 (2.95-6.05)	4.28 (2.60-9.50)	4.18 (2.51-7.90)	4.18 (2.70-6.70)	3.78 (1.50-6.50)
**Sex**	**M** **MC** **F** **FC**	2 15 17 11	4 15 8 18	0 17 0 26	1 13 1 31	1 17 1 19	1 18 1 22
**sCr^b^** **in μmol/L** **in mg/dL**		104.3 (62.8-153.0) 1.2 (0.7-1.7)	112.3 (59.3-175.1) 1.3 (0.7-2.0)	113.2 (66.3-165.3) 1.3 (0.8-1.9)	113.2 (69.0-176.8) 1.3 (0.8-2.0)	202.5 (129.1-519.0) 2.3 (1.5-5.9)	59.24 (33.6-160.9) 0.7 (0.4-1.8)
**USG^c^**		1.050 (1.015-1.060)	1.050 (1.011-1.060)	1.050 (1.021-1.060)	1.046 (1.020-1.060)	1.019 (1.010-1.042)	1.050 (1.016-1.050)

^a^Junior group based on *n* = 30, adult group based on *n* = 34; mature adult group based on *n* = 42; senior group based on *n* = 44; CKD group based on *n* = 36; hyperthyroid group based on *n* = 39.

^b^Junior group based on *n* = 44; hyperthyroid group based on *n* = 41.

^c^Junior group based on *n* = 43; hyperthyroid group based on *n* = 37. Abbreviations: CKD = chronic kidney disease; F = female; FC = female castrated; M = male; MC = male castrated; sCr = serum creatinine; USG = urine specific gravity.

**Figure 1:**
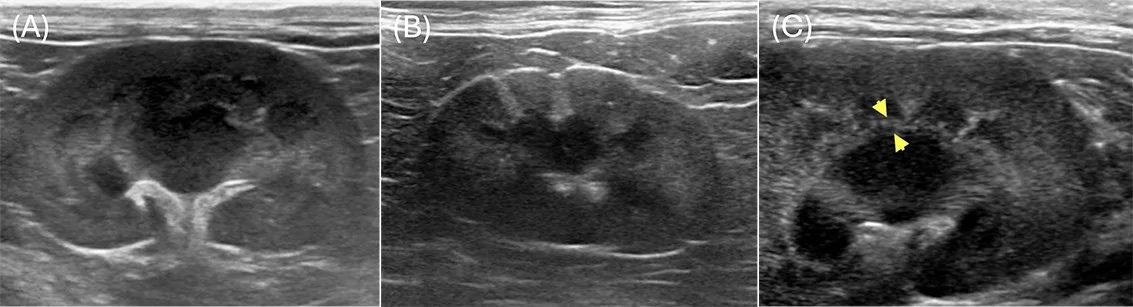
Dorsal renal sonograms of (A) a healthy cat without renal abnormalities, (B) a cat with CKD, and (C) a hyperthyroid cat showing renal abnormalities. The CKD-affected cat shows multiple abnormalities, including irregular renal shape, dystrophic mineralization, segmental cortical lesions, mildly decreased corticomedullary definition, and increased cortical echogenicity. The hyperthyroid cat shows a prominent medullary band sign, indicated between the yellow arrows, making accurate measurement of renal dimensions less straightforward. Abbreviation: CKD = chronic kidney disease.

### Renal dimensions in CKD diagnosis

Chronic kidney disease-affected cats had significantly decreased renal length, RCT, and RMT both in the dorsal and transverse planes (*P* < .05) compared with the entire group of healthy cats, age-comparable healthy seniors, and hyperthyroid cats. The CM ratio of CKD cats was significantly higher compared with age-comparable healthy senior cats in both planes and only in the dorsal plane for all healthy cats, but could not be differentiated from hyperthyroid cats. Renal dimensions in hyperthyroid cats did not differ significantly from those in healthy cats, including the age-comparable senior cats, except for the renal cortex, which appeared to be slightly thicker in the hyperthyroid group compared with the overall healthy group when measured in the dorsal plane (*P* < .05). Table 2 shows the means ± SDs for renal dimensions across the different disease-age groups.

**Table 2:** Overview of the estimated group means and associated SDs of the renal variables (ie, renal length, cortical and medullary thickness, and corticomedullary ratio in the dorsal plane, cortical and medullary thickness, and corticomedullary ratio in the transverse plane) for the following groups of cats: junior, young adult, mature adult, and senior healthy cats, cats with CKD, and nonazotemic hyperthyroid cats. Estimated group-level means were calculated from the mean measurements of left and right kidney of each cat (when available), as measured by the expert. All measurements are expressed in centimeter. Groups with distinct superscripts differed significantly (*P* < .05). Groups sharing 1 or 2 letters in superscript did not significantly differ.

	**Junior (1-2 years)**	**Young adult** **(3-6 years)**	**Mature adult** **(7-10 years)**	**Senior (>11 years)**	***C*KD**	**Hyperthyroid**
	***n* = 45**	***n* = 45**	***n* = 43**	***n* = 46**	***n* = 38**	***n* = 42**
Renal length^*^	3.69 ± 0.30	3.76 ± 0.42	3.81 ± 0.33	3.68 ± 0.44^a^	3.19 ± 0.38^b^	3.85 ± 0.45^a^
**RCT dorsal**	0.60 ± 0.10	0.62 ± 0.10	0.64 ± 0.13	0.65 ± 0.12^a^	0.54 ± 0.13^b^	0.67 ± 0.13^a^
**RMT dorsal**	0.97 ± 0.14	0.97 ± 0.16	1.07 ± 0.25	1.08 ± 0.25^a^	0.80 ± 0.18^b^	1.05 ± 0.20^a^
**CM ratio dorsal**	0.63 ± 0.13	0.67 ± 0.16	0.63 ± 0.15	0.63 ± 0.14^a^	0.69 ± 0.15^b^	0.67 ± 0.18^ab^
RCT^**^ transverse	0.63 ± 0.10	0.65 ± 0.09	0.65 ± 0.12	0.65 ± 0.13^a^	0.59 ± 0.12^b^	0.68 ± 0.12^a^
RMT^**^ transverse	1.09 ± 0.16	1.13 ± 0.21	1.27 ± 0.18	1.22 ± 0.25^a^	0.98 ± 0.18^b^	1.19 ± 0.22^a^
CM ratio^**^ transverse	0.60 ± 0.16	0.60 ± 0.14	0.52 ± 0.10	0.55 ± 0.13^a^	0.61 ± 0.13^b^	0.60 ± 0.15^ab^

^*^Senior group based on *n* = 37.

^**^Junior group based on *n* = 42, young adult group based on *n* = 39; mature adult group based on *n* = 40, senior group based on *n* = 45, and hyperthyroid group based on *n* = 40.

Abbreviations: CKD = chronic kidney disease; CM ratio = corticomedullary ratio; RCT = renal cortical thickness; RMT = renal medullary thickness.

Although decreased renal length as well as a decreased RCT and RMT were indicative of the presence of CKD, none of the measurements distinguished among the different IRIS stages of disease. Based on the 38 CKD-affected cats, scatter diagrams were generated with sCr on the X-axis and each of the renal dimensions on the Y-axis showing the evolution of the ultrasound-measured dimensions in relation to disease severity (Figure 2A-C). Overall, linear association between sCr and renal dimensions was weak (with coefficients of determination [*R*^2^] ranging from 0.01 to 0.08), indicating that only a small portion of the variance in ultrasound-measured renal dimensions could be explained by CKD severity.

**Figure 2A:**
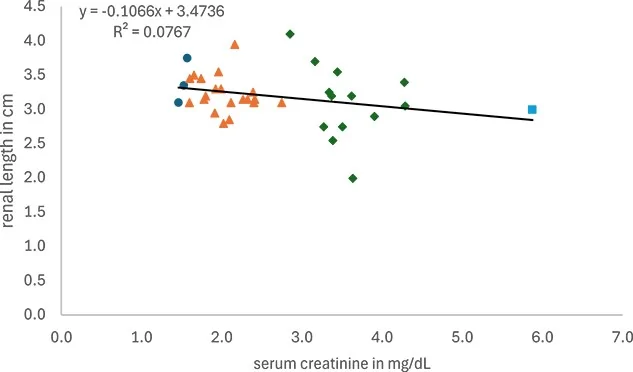
Based on the group of *n* = 37 cats diagnosed with CKD, the scatter plot illustrates the variation in renal length in relation to the disease severity. Serum creatinine concentrations on the X-axis are plotted against the mean renal length of both kidneys estimated in the longitudinal scan plane on the Y-axis. Cats at different IRIS stages are represented by distinct symbols: IRIS stage 1 (*n* = 3) by dots; IRIS stage 2 (*n* = 20) by triangles; IRIS stage 3 (*n* = 13) by diamonds; and IRIS stage 4 (*n* = 1) by a square. The coefficient of determination (*R*^2^) of 0.08 indicates a weak linear relationship between renal length and disease severity. Abbreviations: CKD = chronic kidney disease; IRIS = International Renal Interest Society.

**Figure 2B:**
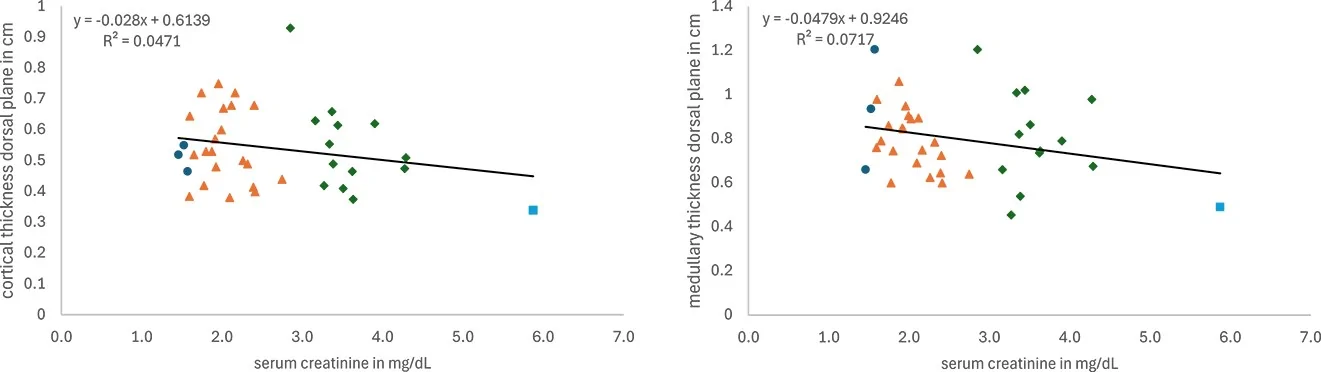
Based on the group of *n* = 38 CKD-diagnosed cats, the scatter plots illustrate the variation in RCT and RMT in the dorsal plane in relation to the disease severity. Serum creatinine concentrations on the X-axis are plotted against the mean RCT (left) and RMT (right) of left and right kidney measured in the dorsal plane on the Y-axis. Cats at different IRIS stages are represented by distinct symbols: IRIS stage 1 (*n* = 3) by dots; IRIS stage 2 (*n* = 21) by triangles; IRIS stage 3 (*n* = 13) by diamonds; and IRIS stage 4 (*n* = 1) by a square. Coefficients of determination (*R*^2^) range from 0.05 to 0.07, indicating a weak linear relationship between the RCT, RMT, and disease severity. Abbreviations: CKD = chronic kidney disease; IRIS = International Renal Interest Society; RCT = renal cortical thickness; RMT = renal medullary thickness.

**Figure 2C:**
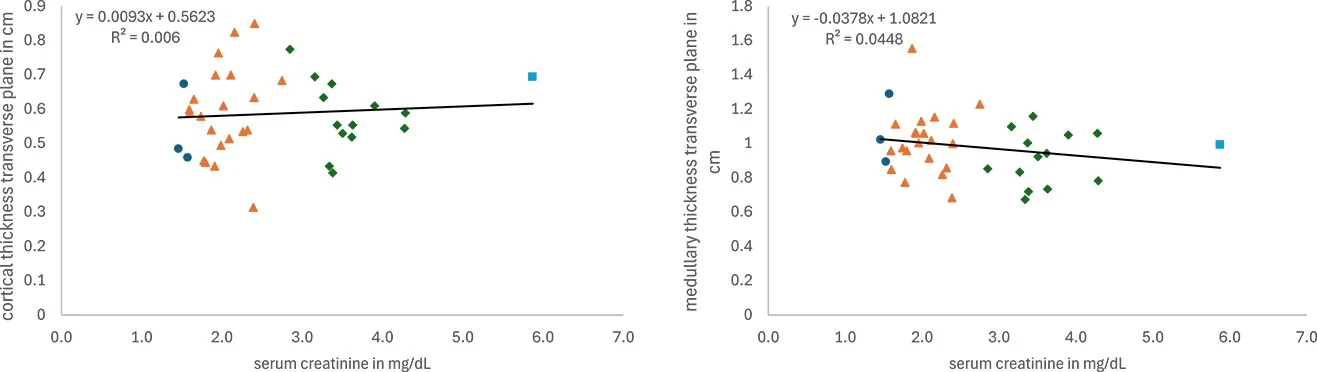
Based on the group of *n* = 38 CKD-diagnosed cats, the scatter plots illustrate the variation in RCT and RMT in the transverse plane in relation to the disease severity. Serum creatinine concentrations on the X-axis are plotted against the mean RCT (left) and RMT (right) of left and right kidney measured in the transverse plane on the Y-axis. Cats at different IRIS stages are represented by distinct symbols: IRIS stage 1 (*n* = 3) by dots; IRIS stage 2 (*n* = 21) by triangles; IRIS stage 3 (*n* = 13) by diamonds; and IRIS stage 4 (*n* = 1) by a square. Coefficients of determination (*R*^2^) range from 0.01 to 0.05, indicating a weak linear relationship between the RCT, RMT, and disease severity. Abbreviations: CKD = chronic kidney disease; IRIS = International Renal Interest Society; RCT = renal cortical thickness; RMT = renal medullary thickness.

The ROC curves were constructed (Figure 3A-D) to further evaluate the ability of renal dimensions to discriminate between kidneys of CKD-affected cats and those of age-comparable senior healthy cats. Based on the area under the curve (AUC) (Table 3), considerable diagnostic accuracy was observed for renal length, and RMT (measured in both planes) although CIs were wide with a lower limit that affected diagnostic accuracy. Renal cortical thickness measured in both planes and derived CM ratios showed fair to poor discriminatory performance, with the lower end of the 95% CI near 0.50 for the CM ratios. Visual inspection of RCT and RMT ROC plots suggested slightly better diagnostic accuracy when cortex and medulla were measured in the dorsal scan plane. The results demonstrate that renal length, and RMT (especially in the dorsal plane) may be valuable to diagnose CKD. For renal length, the optimal cut-off was set at 3.33 cm, yielding a sensitivity of 70% and a specificity of 80%. For dorsally measured RMT, the optimal cut-off was set at 0.99 cm, yielding a sensitivity of 87% and specificity of 67%. Values below (or equal to) this cut-off were considered indicative of CKD (Table 3).

**Figure 3:**
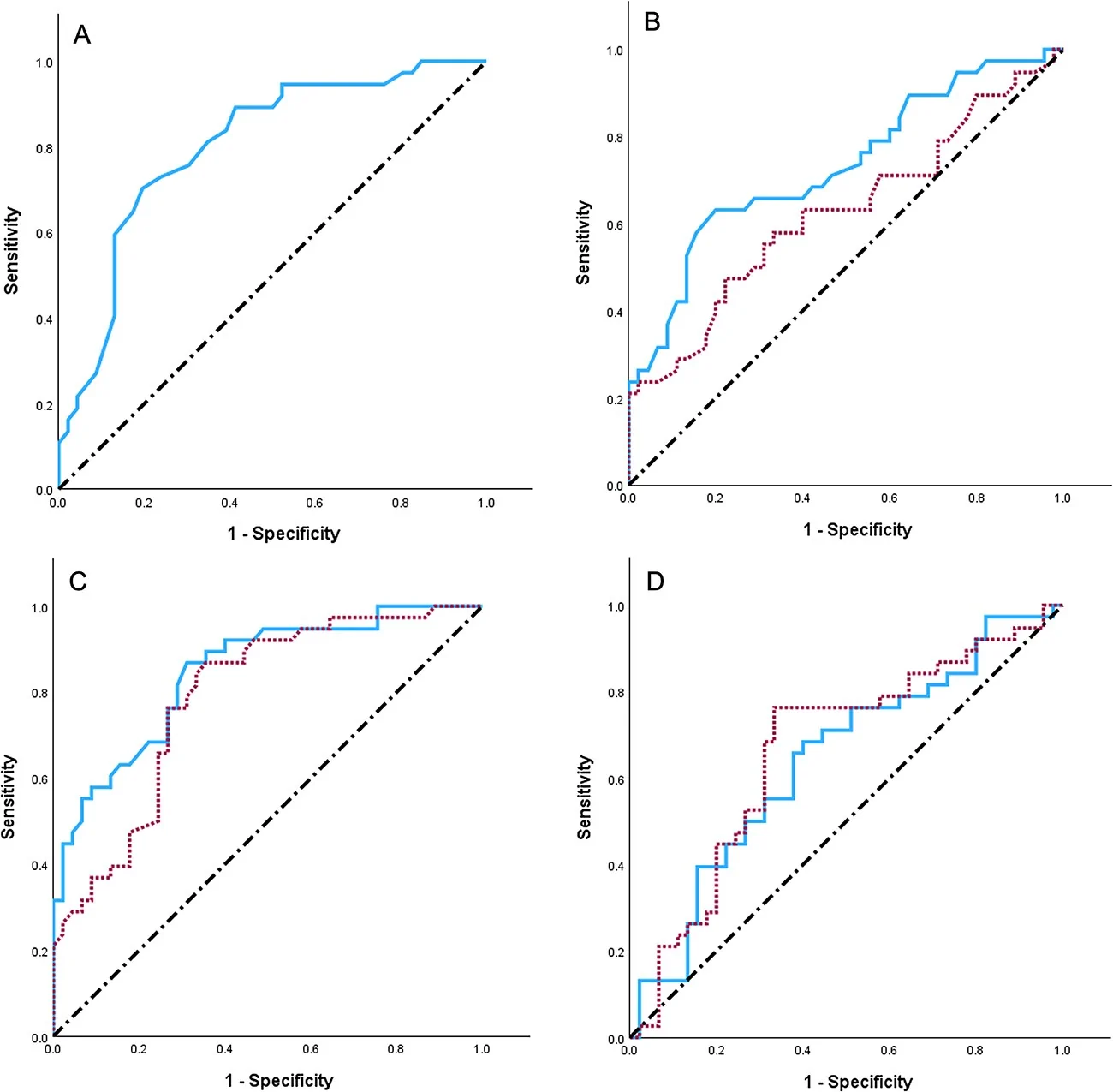
Receiver operating characteristic (ROC) plots for ultrasound-based renal dimensions: (A) renal length, (B) RCT, (C) RMT, and (D) calculated CM ratios. By plotting 1 − specificity (X-axis) against sensitivity (Y-axis) for a range of plausible cut-off values, ROC curves were generated to evaluate diagnostic accuracy (expressed as the AUC) of each renal dimension in distinguishing cats with CKD from healthy senior cats. The ROC plots of RCT, RMT, and calculated CM ratios each contain 2 ROC curves, thereby showing the measurements of the dorsal (solid curve) and the transverse scan plane (dotted curve). Table 3 further details the AUC (and corresponding 95% CIs), optimal cut-off and corresponding specificity and sensitivity for each renal dimension. Abbreviations: AUC = area under the curve; CKD = chronic kidney disease; CM = corticomedullary; RCT = renal cortical thickness; RMT = renal medullary thickness.

**Table 3:** For all ultrasound-based renal dimensions (ie, renal length, RCT, and RMT in the dorsal and transverse scan plane, and the calculated CM ratios in the dorsal and transverse scan plane); the AUC (and corresponding 95% CI), the optimal cut-off value (expressed in centimeter) and the corresponding sensitivity and specificity are reported to evaluate the diagnostic performance in distinguishing cats with chronic kidney disease (*n* = 38) from age-matched healthy senior cats (*n* = 46). For all cats (*n* = 84) the mean measurements of left and right kidney, as measured by the expert, were used. For renal length, RCT (dorsal and transverse) and RMT (dorsal and transverse), values less than or equal to the cut-off value indicate CKD. For both CM ratios values higher than or equal to the cut-off value indicate CKD.

	**AUC (95% CI)**	**Optimal cut-off**	**Sensitivity**	**Specificity**
**Renal length^a^**	0.80 (0.71-0.90)	≤3.33	0.70	0.80
**RCT dorsal**	0.73 (0.62-0.84)	≤0.56	0.63	0.80
**RCT transverse^b^**	0.63 (0.50-0.75)	≤0.56	0.47	0.78
**RMT dorsal**	0.84 (0.76-0.92)	≤0.99	0.87	0.67
**RMT transverse^b^**	0.79 (0.69-0.89)	≤1.13	0.87	0.64
**CM ratio dorsal**	0.64 (0.52-0.76)	≥0.64	0.68	0.59
**CM ratio transverse^b^**	0.67 (0.55-0.79)	≥0.56	0.76	0.67

a Based on *n* = 83: *n* = 37 for CKD group and *n* = 46 for senior healthy group.

b Based on *n* = 83: *n* = 38 for CKD group and *n* = 45 for senior healthy group.

Abbreviations: AUC = area under the receiver operating characteristic curve; CKD = chronic kidney disease; CM ratio = corticomedullary ratio; RCT = renal cortical thickness; RMT = renal medullary thickness; AUC = area under receiver operating characteristic curve.

### Influence of demographic variables and kidney laterality on renal dimensions

Renal length, RCT (only when measured in the transverse plane), RMT, and the derived CM ratios differed significantly between the left and right kidneys (*P* < .05). More specifically, both renal length and the transversely measured renal cortex were smaller in the left kidney, whereas the renal medulla was larger on the left side. As presented in Table S3, left–right differences were minor in magnitude (typically a few millimeters).

As a result, CM ratios calculated from the left kidney were lower. When analyzed within the health groups, left–right difference in renal length was no longer significant. In contrast, left–right asymmetry in transverse RCT, RMT, and CM ratios remained significant in healthy cats but were no longer significant in the diseased groups, except for the transverse CM ratio in the CKD group. However, the smaller subsample sizes may have limited ability to detect some notable asymmetries.

Body weight had a small but significant positive effect on renal length (β = 0.17 ± 0.02, *P < .*05), RCT (β = 0.04 ± 0.01, *P < .*05 in both dorsal plane and transverse plane), and RMT (β = 0.05 ± 0.02, *P < .*05 in dorsal plane; β = 0.06 ± 0.02, *P < .*05 in transverse plane) in healthy cats. However, the derived CM ratios were not significantly affected by weight. Age had a significant impact on RMT, but not on renal length, or RCT. In healthy cats, RMT increased slightly with age (β = 0.01 ± 0.004 in dorsal plane; β = 0.02 ± 0.004 in transverse plane), which was reflected in a significantly lower CM ratio only transversely (*P* < .05).

Sex-associated differences in renal dimensions were observed (*P* < .05), most notably for renal length. Intact and neutered males exhibited significantly higher renal length compared with intact and neutered females. Being neutered made no difference in terms of renal length. Neutered males also had thicker cortex compared with neutered females, and thicker medulla compared with intact females (*P* < .05). In female cats, gonadal status made a difference for RMT because the renal medulla was found to be thicker in neutered females (in the transverse plane only). Because sex-related variation was generally minimal, the derived CM ratios did not significantly differ between sexes (Table S4).

### Effects of inter- and intraobserver variability on renal dimensions

Overall, interobserver agreement varied considerably across the renal dimensions, with good agreement (ICC ≥ 0.75) for renal length, moderate agreement (ICC ≥ 0.50) for RMT in both planes, but poor agreement (ICC < 0.50) for RCT and CM ratios in both planes. Compared with the less experienced observer, the expert scored renal length (mean of paired differences between observers [$\overline{d}$] ± SD of paired differences [SD_d_]: −0.03 ± 0.22 cm), and the RCT ($\overline{d}$ ± SD_d_: −0.04 ± 0.14 cm dorsally; $\overline{d}$ ± SD_d_: −0.03 ± 0.15 cm transverse) slightly lower and the RMT ($\overline{d}$ ± SD_d_: 0.05 ± 0.18 cm dorsally; $\overline{d}$ ± SD_d_: 0.01 ± 0.22 cm transverse) slightly higher. The systematic bias was overall small but considerable dispersion around the bias was observed for all renal dimensions, resulting in wide 95% LoA and indicating substantial variability in the measurement differences between observers (Figure 4). Interobserver agreement in renal dimensions also differed according to health status, with the lowest agreement being observed in the healthy group for most renal dimensions. Table 4a provides a summary of ICC’s for each renal dimension in the total study sample and according to health status.

**Figure 4:**
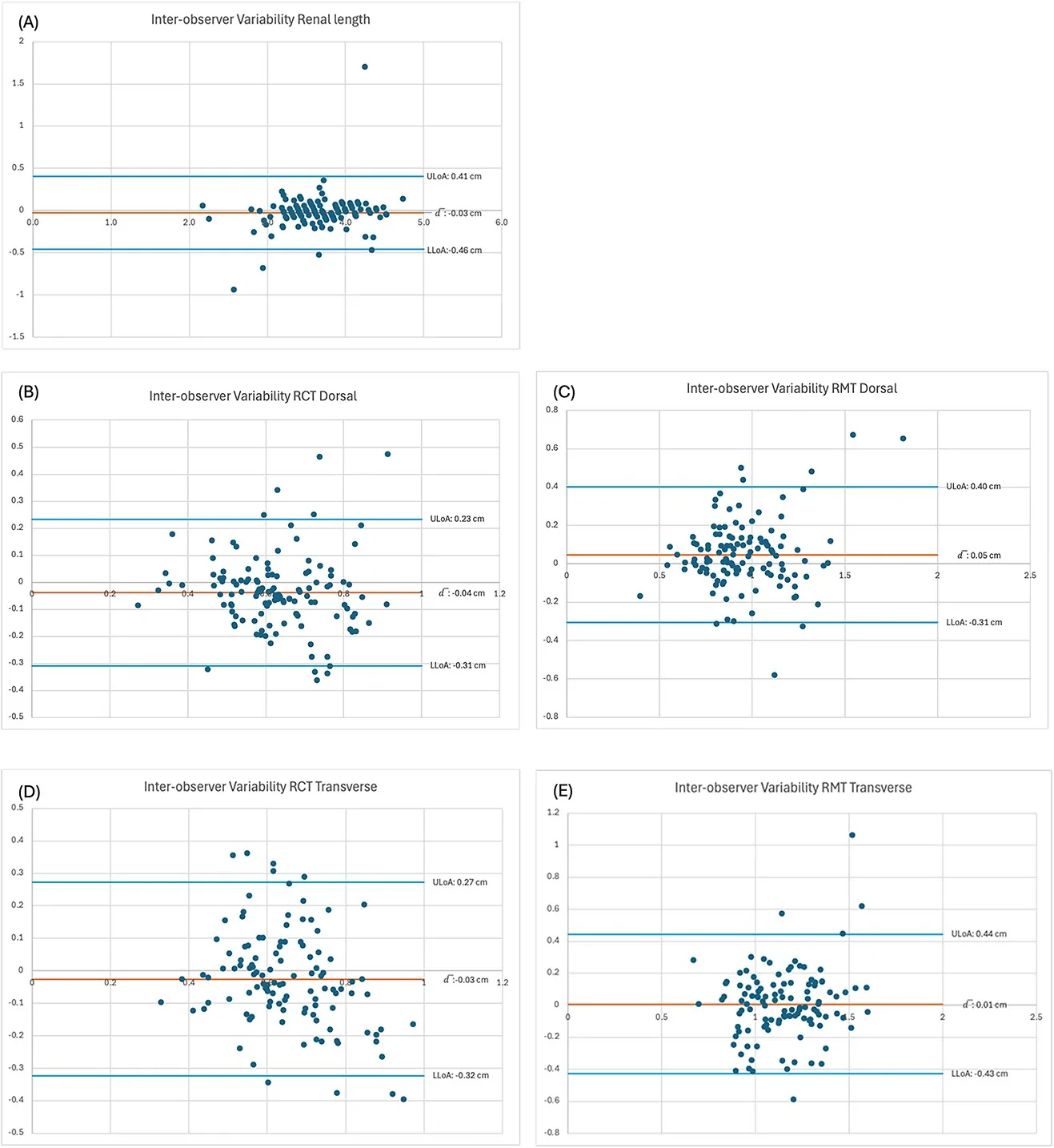
Bland–Altman plots illustrating the interobserver variability for ultrasound-based renal measurements: (A) renal length, (B) RCT, and (C) RMT measured in the dorsal scan plane; (D) RCT and (E) RMT measured in the transverse scan plane. The plots display the mean of the paired measurements by observers 1 and 2 (the X-axis), against the differences between the paired measurements of both observers (the Y-axis). For observer 2, the mean of the replicate measurements was used. The middle horizontal line represents the mean of the paired differences between observers ($\overline{d}$). The upper and lower horizontal lines represent the 95% LoA, calculated as $\overline{d}$ ± 1.96 × SD of the differences (SD_d_). Abbreviations: LoA = limits of agreement; RCT = renal cortical thickness; RMT = renal medullary thickness.

**Table 4a:** Summary of interobserver agreement for ultrasound-based renal dimensions (ie, renal length, RCT, RMT, and derived CM ratios in the dorsal and transverse scan planes). The data are based on the total number of paired measurements (*n* = 130), and for subgroups of cats stratified by health status. Agreement is expressed as ICCs. ICC: < 0.50: poor; ICC: 0.50— < 0.75: satisfactory; ICC  ≥ 0.75: good.

	**Total**	**Healthy**	**CKD**	**Hyperthyroidism**
	***n* = 130**	***n* = 89**	***n* = 21**	***n* = 20**
**Renal length^a^**	0.88	0.84	0.79	0.98
**RCT dorsal^b^**	0.50	0.37	0.55	0.73
**RCT transverse^c^**	0.43	0.35	0.75	0.30
**RMT dorsal^b^**	0.69	0.62	0.71	0.85
**RMT transverse^c^**	0.56	0.42	0.58	0.78
**CM ratio dorsal^b^**	0.41	0.35	0.55	0.64
**CM ratio transverse^c^**	0.19	0.16	0.33	0.21

a Based on *n* = 128 paired measurements; *n* = 87 in healthy group, *n* = 21 in CKD group, and *n* = 20 in hyperthyroid group.

b Based on *n* = 124 paired measurements; *n* = 86 in healthy group, *n* = 19 in CKD group, and *n* = 19 in hyperthyroid group.

c Based on *n* = 114 paired measurements; *n* = 78 in healthy group, *n* = 16 in CKD group, and *n* = 20 in hyperthyroid group.

Abbreviations: CKD = chronic kidney disease; CM ratio = corticomedullary ratio; ICCs = intraclass correlation coefficients; RCT = renal cortical thickness; RMT = renal medullary thickness.

**Table 4b:** Summary of intra-observer agreement for ultrasound-based renal dimensions (ie, renal length, RCT, RMT, and derived CM ratios in the dorsal and transverse scan planes). The data are based on the total number of replicate measurements (*n* = 130), and for subgroups of cats stratified by health status. Agreement is expressed as ICCs. ICC: < 0.50: poor; ICC: 0.50- < 0.75: satisfactory; ICC ≥ 0.75: good.

	**Total**	**Healthy**	**CKD**	**Hyperthyroidism**
	***n* = 130**	***n* = 89**	***n* = 21**	***n* = 20**
**Renal length^a^**	0.95	0.99	0.80	0.99
**RCT dorsal^b^**	0.96	0.96	0.95	0.98
**RCT transverse^c^**	0.98	0.98	0.95	0.99
**RMT dorsal^b^**	0.97	0.98	0.90	0.98
**RMT transverse^c^**	0.98	0.99	0.90	0.99
**CM ratio dorsal^b^**	0.98	0.98	0.98	0.99
**CM ratio transverse^c^**	0.98	0.97	0.97	0.99

a Based on *n* = 129 paired measurements; *n* = 88 in healthy group, *n* = 21 in CKD subgroup, and *n* = 20 in hyperthyroid group.

b Based on *n* = 125 paired measurements; *n* = 87 in healthy group, *n* = 19 in CKD group, and *n* = 19 in hyperthyroid group.

c Based on *n* = 118 paired measurements; *n* = 79 in healthy group, *n* = 19 in CKD group, and *n* = 20 in hyperthyroid group. Abbreviations: CKD = chronic kidney disease; CM ratio = corticomedullary ratio; ICCs = intraclass correlation coefficients; RCT = renal cortical thickness; RMT = renal medullary thickness.

With the intraobserver ICCs consistently > 0.95; excellent repeatability for all renal dimensions was observed when scored by the same observer (Table 4b). Based on BA analysis, all renal dimensions showed minimal mean differences between replicate measurements (range, −0.003 to 0.002 cm), narrow dispersion, and acceptable 95% LoA (Figure 5). The intraobserver agreement did not vary significantly with cat health status.

**Figure 5:**
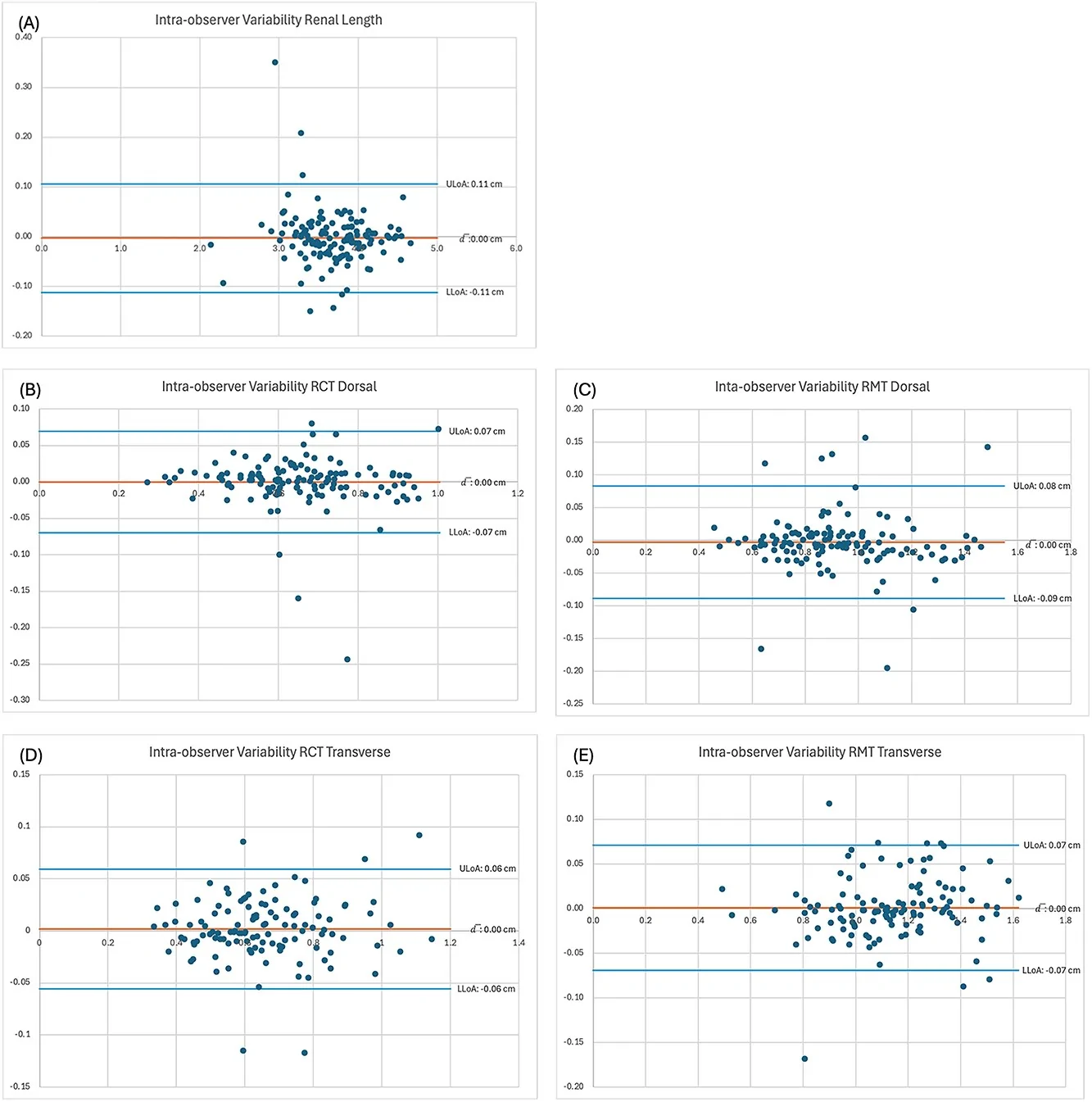
Bland–Altman plots illustrating the intraobserver variability for ultrasound-based renal measurements: (A) renal length, (B) RCT, and (C) RMT measured in the dorsal scan plane; and (D) RCT and (E) RMT measured in the transverse plane. The plots display the mean of the replicate measurements by observer 2 (X-axis), against the difference between the replicate measurements of observer 2 (Y-axis). The middle horizontal line represents the mean of paired differences between replicate measurements by the novice ($\overline{d}$). The upper and lower horizontal lines represent the 95% LoA, calculated as $\overline{d}$ ± 1.96 × SD of the differences (SD_d_). Abbreviations: LoA = limits of agreement; RCT = renal cortical thickness; RMT = renal medullary thickness.

## Discussion

Our retrospective study aimed to assess the diagnostic value of ultrasound-measured renal dimensions in cats with CKD, by evaluating the influence of demographic variables, within-subject renal differences, and intra- and interobserver measurement variation. The results identified significant differences in renal length, RCT and RMT (in dorsal and transverse scan planes) between CKD-affected cats and both nonazotemic hyperthyroid and clinically healthy control cats (independent of age). Cats with CKD had a decreased renal length, along with a slightly thinner cortex and thinner medulla compared with the other groups. Group differences were generally small, but renal length and RMT showed considerable diagnostic potential. Conversely, CM ratios calculated from the RCT and RMT were only weakly associated with renal function, which limits their diagnostic utility. None of the predefined renal dimensions reliably discriminated among the different IRIS stages of CKD severity. Most renal dimensions were subject to small left–right kidney asymmetry, which was primarily observed in healthy cats and consequently reached statistical significance for the entire study sample.

In healthy cats, the demographic variables body weight, and to a lesser extent sex (gonadal status) and age further contributed to the biological variability of renal dimensions. We also found that renal measurements were markedly affected by interobserver variability, emphasizing the importance of ultrasonographic expertise.

We expected that the CKD-affected cats would predominantly manifest decreased RCT, given that CKD histopathologically is characterized by chronic tubulointerstitial nephritis and fibrosis,^2^^,^^14^^,^^15^ primarily affecting the renal cortex. Our results did confirm the presence of renal cortical thinning in CKD-diagnosed cats. Compared with both healthy and hyperthyroid control cats, CKD-affected cats had relatively small absolute decreases in RCT (range, 0.06-0.13 cm), but the corresponding percentage decreases (range, 9.14%-19.52%) were nevertheless substantial. Regardless, the linear association between RCT and sCr was weak, and RCT had only fair to poor discriminatory power, limiting its clinical utility as a diagnostic marker for CKD in cats. Unexpectedly, we observed significant and more pronounced renal medullary thinning in CKD-affected cats compared with healthy and hyperthyroid controls, with absolute decreases of 0.20-0.28 cm, corresponding to relative decreases of 17.23%-26.25%. Renal medullary thickness had adequate diagnostic performance (particularly in the dorsal scan plane) and outperformed other renal dimensions, which emphasizes its potential as a screening tool for CKD in cats. Because cortical thinning was counterbalanced by the more pronounced medullary thinning, resulting CM ratios of CKD cats were slightly increased, but these changes were too small to provide adequate diagnostic power. Consequently, the CM ratio appeared unreliable as a diagnostic marker for CKD in cats.

The lack of diagnostic potential of the CM ratio observed in our study contrasts with a similar but smaller study (19 healthy and 30 cats with CKD), in which substantial cortical thinning was observed in CKD-affected cats without significant medullary involvement, resulting in a significantly decreased CM ratio from early IRIS stages onward.^2^ Absolute transverse RCT results were generally lower, particularly in CKD cats in which the cortex appeared substantially thinner (0.38 vs 0.59 cm) with relative decreases from healthy controls ranging from 26% to 31.42%, which were more pronounced than in our study. The absolute transverse RMT results in healthy controls were comparable across studies, but whereas we observed significant medullary thinning in CKD cats, the previous study reported only a nonsignificant decrease in RMT compared to the healthy group (range, 5.43%-8%).^2^ Even after correcting absolute measurement differences between both studies, discrepancies in relative changes of CKD from healthy controls across the studies remained. Several reasons may explain the inconsistent findings. An accurate distinction between RCT and RMT mainly relies on proper identification of arcuate vessels in the CM junction. Because of changes in cortical and medullary echogenicity caused by pathologic alterations in renal architecture, the CM junction may become less distinct in cats with CKD,^1^^,^^16^ making identification of arcuate vessels challenging. In our study, approximately 70% of CKD-diagnosed cats had mildly to severely decreased CM definition, potentially complicating accurate delineation of renal cortex and medulla and contributing to substantial interobserver variation, as reflected in our data. Hyperechogenicity of the renal medulla was not exclusive to the CKD cats in our study, but was observed in over half of them and appeared overrepresented in this group, which mainly consisted of cats in IRIS stages 2 and 3. This observation suggests that tubulointerstitial inflammation and fibrosis may reach beyond the cortex, involving the outer medulla and contributing to medullary thinning, even in earlier stages of the condition. Ultrasound-detected medullary changes associated with CKD in cats have not yet been extensively documented.^3^ However, medullary scarring has been reported in the early stages of CKD in cats^15^ and earlier computed tomography (CT)-based research indicated that decreases in RMT occur concurrently with decreases in RCT,^17^ a finding consistent with our data.

In addition to the previously discussed CM changes and medullary hyperechogenicity, most other renal abnormalities were predominantly present in the CKD-affected group, potentially further complicating accurate delineation and measurement of RCT and RMT.

Biological variation in RCT, RMT, and the resulting CM ratio generally was small. The left kidney displayed a slightly thicker medulla, marginally thinner cortex and smaller CM ratio compared to the right kidney, findings consistent with earlier research in cats.^9^ With regard to demographic factors, RMT increased significantly with both age and body weight, whereas RCT increased significantly with body weight only, although these effects were small. Cortices tended to be thicker in male than in female cats, reaching significance only between neutered males and neutered females (0.06 cm, *P* < .05). Similarly, medullary thickness had limited sex-related differences, significant only between neutered males and intact females (0.13-0.20 cm, *P* < .05). Demographic and individual factors contribute to variation in CM thickness and CM ratios, but the magnitude of the effects is rather small. Some of the observed variation also may be attributable to technical factors or artifacts. Overall, the impact on clinical interpretation of ultrasonographic measurements is thought to be limited.

Evaluation of renal length is a routine part of abdominal ultrasonography and traditionally has been used as a surrogate for renal function.^3^^,^^4^^,^^18^ However, recent studies have shown that normal renal length may vary widely in cats (range, 2.98-5.09 cm)^7^^,^^18^ and only a small percentage of cats with CKD have abnormal renal length outside of this range.^18^ The heterogeneous nature of the condition results in an overlap in renal length between healthy and diseased cats, limiting its usefulness as a surrogate marker of CKD in cats, and explaining its poor correlation with the renal function. In both human and feline medicine, renal length generally is considered inferior to RCT as an indicator of CKD.^2^^,^^4^^,^^5^^,^^18^ Contrary to our expectations (and partly because of the suboptimal performance of RCT), renal length in our study proved to be a more valuable indicator than RCT for identifying CKD in cats. Compared with (age-comparable) healthy and nonazotemic hyperthyroid cats, the CKD cats featured a marked decrease in renal length, and this variable had considerable discriminative power, comparable to that of RMT. With a cut-off value of 2.9 cm (the lower limit of the reported reference interval) only 19% of CKD-affected cats would be recognized as such, results comparable with previous findings.^18^ However, using our slightly higher optimal cut-off of 3.33 cm, we achieved moderate sensitivity and acceptable specificity. Our large and statistically significant between-group differences were facilitated by the small within-group variability, a finding that contrasts earlier studies in cats that reported much higher intragroup variation in renal length.^2^^,^^18^ A possible explanation for the observed low within-group variability is our choice to use mean values from the left and right kidney, which may have minimized possible large differences between both kidneys.

Moreover, by using this averaging approach, we a priori assumed that physiological left–right asymmetry would be clinically negligible and that metabolic disease or demographic variables would have similar influences on both kidneys. Although no clinically relevant left–right differences were detected within the CKD or hyperthyroid groups, the heterogeneous presentation of CKD makes individual renal asymmetry plausible. Analyzing kidneys separately might therefore have offered additional diagnostic value in detecting CKD-related changes.

Nevertheless, the left–right differences in renal length observed in our study sample were small (1 ± 0.4 mm) and, despite reaching statistical significance, they are likely explained by technical factors.^9^ Feline kidneys, especially the left, are mobile and easily displaced by transducer pressure whereas right-sided renal imaging is complicated by the cranial position of the right kidney partly under the costal arch, and its proximity to the descending duodenum and ascending colon, which often contain gas.^9^^,^^19^ Consequently, measurement differences between both kidneys are of limited clinical relevance. In addition to the technical factors, biological variation further contributes to renal length. Consistent with earlier research,^7^^,^^8^ body weight and sex were significantly associated with renal length in healthy cats; males (regardless of neuter status) and heavier cats tended to have larger kidneys. Overall, renal length increased by 0.17 cm for every kilogram of body weight. Because body weight and sex are related, individual contributions to renal length cannot be fully separated, but they likely account for a substantial proportion of the physiological variability within the reference interval. In our dataset of healthy cats evenly distributed across life stages, a subtle upward trend in renal length was observed until the senior life stage, where a decrease in renal length occurred, a pattern previously reported in studies of humans. However, we did not confirm a statistically significant effect of age on renal length.

Despite excellent intraobserver repeatability for all renal dimensions, the interobserver agreement was overall considerably lower. Longitudinally measured renal length was the only variable that demonstrated excellent agreement between observers, emphasizing its clinical robustness in cats with CKD. Renal cortical thickness was less consistently scored between both observers than RMT. The compounded variability when combining these measurements likely accounts for the poor reproducibility of the CM ratios. Moreover, dorsal measurements consistently had higher interobserver agreement, suggesting this plane allows clearer identification of the needed landmarks (ie, arcuate vessels, renal crest), making dorsal renal images preferable for measuring renal dimensions. Compared to diseased cats, healthy cats often exhibited the lowest agreement between observers, an observation for which the underlying cause remains unclear. Our findings differ from a previous study,^2^ that described overall satisfactory to good interobserver agreement (except for the derived CM ratios), in particular for transverse measurements. These observers had a substantial and comparable imaging experience, which likely contributed to the higher reproducibility. In our study, renal measurements were performed by 2 observers with markedly different levels of expertise (an imaging novice vs imaging specialist), which inevitably increased interobserver variability. This finding underscores the importance of sufficient ultrasonography training, both in acquiring images at correct mid-kidney level and reviewing them. Our findings raise concerns about the broader applicability of ultrasound-based renal dimensions during routine CKD screening and follow-up in cats, particularly in general practice settings where such imaging expertise may not readily be available.

### Limitations

The main limitations of our study are primarily related to its retrospective study design. Renal ultrasonographic evaluation was performed at a single time point only. A longitudinal approach involving repeated renal imaging of the same cats over time (eg, from the healthy baseline to the hyperthyroid state or through progressive CKD stages) would have provided more robust evidence regarding the impact of disease and its progression on renal dimensions, while minimizing the confounding effect of interindividual variability. In addition, ultrasonographic images were collected over a 12-year period by multiple imagers using different ultrasound machines. Although images were acquired using a standardized scanning technique, these factors likely affected image consistency and image quality, making accurate evaluation of renal length, RCT, and RMT challenging in some cats. This factor likely contributed to the observed inter- and intraobserver variability in measurements. Another limitation relates to the assessment of renal health status. Classification was based on routine serum and urine biomarkers, with symmetric dimethyl arginine (SDMA) not being available for all cats and longitudinal trends in renal biomarkers not consistently evaluated. Consequently, early-stage (nonazotemic) CKD may have remained undetected in some cases, potentially resulting in misclassification of affected cats as healthy. Furthermore, all hyperthyroid cats were untreated at the time of ultrasonographic evaluation. It must be acknowledged that occult CKD may have been present in some hyperthyroid cats, which could have influenced observed intergroup differences in renal dimensions. Finally, the limited number of CKD-affected cats inherently decreased the precision of statistical estimates, resulting in wider CI’s and decreased statistical power to detect significant differences. This possibility should be taken into account when interpreting the results. The small number of cats with IRIS stages 1 and 4 disease limited our ability to evaluate the relationship between renal dimensions and disease severity across the full range of IRIS stages. Future studies including larger numbers of CKD-affected cats are warranted to further refine the diagnostic value of renal ultrasonographic measurements in cats with CKD.

## Conclusion

Demographic variables (eg, age, weight, and sex) and left–right kidney asymmetry contributed to biological variation in renal dimensions, but this variation appeared to have little clinical relevance. After accounting for age-related renal changes, ultrasound-based renal dimensions successfully differentiated CKD-affected cats from healthy controls whereas hyperthyroidism did not consistently influence the measurements. Among all of the measured dimensions, renal length and RMT were significantly decreased in cats with CKD and emerged as the most effective variables to distinguish cats with CKD from unaffected cats, demonstrating sufficient diagnostic power. Unfortunately, renal dimensions were unable to identify cats with variable degrees of disease severity. Moreover, the level of ultrasonographic expertise plays an essential role in obtaining reliable measurements.

## Supplementary Material

Supplementary_material_aalag274

## Figures and Tables

**Figure A1:**
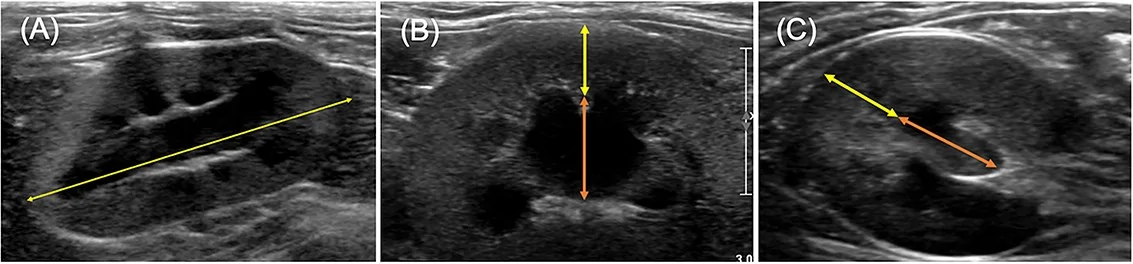
Ultrasonographic images illustrating the measurement of renal dimensions. (A) Renal length measured in the longitudinal plane (double-headed arrow). (B) Dorsal and (C) transverse renal images showing renal cortical thickness (outer double-headed arrow) and renal medullary thickness (inner double-headed arrow).

## Off-label antimicrobial declaration

The authors declare no off-label use of antimicrobials.

## Institutional animal care and use committee or other approval declaration

The authors declare no institutional animal care and use committee or other approval was needed.

## Appendix: Materials and methods

### Population

The study was conducted at the small animal clinic; Faculty of Veterinary Medicine; Ghent University. Medical records of client-owned cats that had undergone abdominal ultrasonography between 2010 and 2022 were reviewed. Cats were retrospectively retained for the study if they met specific health criteria and if sonograms of the kidneys (preferably longitudinal, dorsal, and transverse) were available for review.

Cats were included if they were healthy, or diagnosed with either nonazotemic untreated hyperthyroidism or CKD at the time of renal ultrasonography. Health status of cats was assessed based on the combination of a medical history, physical examination, comprehensive blood examination (including CBC and serum biochemistry profile with total thyroxine [TT4]), urinalysis (comprising urinary specific gravity [USG], urinary pH, urinary protein: creatinine ratio [UPC], sediment examination, and bacterial culture), systolic blood pressure measurement by the Doppler ultrasonic technique, and abdominal ultrasonography.

Cats were considered healthy if no clinically relevant abnormalities were detected in the history, physical examination, blood and urine analyses, and abdominal ultrasonography. Incidental, nonspecific renal ultrasonographic findings in the absence of clinical, biochemical, or urinalysis abnormalities were not regarded as clinically relevant. Based on their age, healthy cats were grouped into 4 life stages largely in agreement with the 2021 American Animal Hospital Association (AAHA) and the American Association of Feline Practitioners (AAFP) feline life stage guidelines: junior (1-2 years), young adult (3-6 years), mature adult (7-10 years), and senior (≥11 years).^10^

Diagnosis of azotemic CKD was based on compatible clinical signs and the presence of renal azotemia; defined as a serum creatinine (sCr) concentration above the laboratory upper reference limit in combination with inadequately concentrated urine (USG < 1.035). Nonazotemic CKD (ie, International Renal Interest Society [IRIS] stage 1 and early stage 2) was defined as sCr within the laboratory reference interval (RI) with some other renal abnormality, which might include: persistent inadequate USG (<1.035) without identifiable nonrenal cause, persistent renal proteinuria (UPC > 0.4), an upward trend in sCr (≥30%) or SDMA (≥50%) within the RI or persistently increased SDMA (>14 μg/dL but < 18 μg/dL) or some combination of these. Persistence of abnormalities or an upward trend in biomarkers was confirmed on repeated testing within 2.5-3.5 months. Abnormalities observed on renal ultrasonography were not considered as the sole diagnostic criterion to avoid any interference with study outcomes. Once CKD was diagnosed, cats were staged according to the IRIS staging guidelines.^11^

Diagnosis of hyperthyroidism was based on a combination of compatible clinical signs, serum TT4 concentration above the upper laboratory reference limit, and increased pertechnetate uptake in one or both thyroid glands on a pertechnetate scan performed using standard methodologies.^12^ Cats with concurrent azotemic CKD were excluded.

### Ultrasonographic procedure

Abdominal ultrasonography was performed by a Diplomate of the European College of Veterinary Diagnostic Imaging (ECVDI) or a supervised ECVDI Resident, following a standardized protocol and mostly without sedation. The ventrolateral aspect of the abdomen was clipped and coupling gel was applied to the skin. If clipping was not allowed, the hair was separated, and visibility was ensured by applying water, alcohol, and coupling gel. Cats were manually restrained in dorsal recumbency. All examinations were conducted using a linear transducer (12-5 MHz) on a dedicated ultrasound machine: IU22 and EPIQ 7 (Philips Healthcare, Bothell, WA, USA) or LOGIQ 7 (GE Medical Systems, Milwaukee, WI, USA). Longitudinal, dorsal and transverse images of the kidneys were obtained using an acknowledged scanning technique^12^ and the saved images were evaluated for the study.

### Observers and measurements

Two observers evaluated the renal sonograms. The first observer was an ECVDI diplomate with extensive expertise in renal ultrasonography (ie, the expert) and reviewed images of the kidneys of all included cats a single time. The second observer was a small animal veterinarian with minimal experience in medical imaging (ie, the novice), who reviewed one randomly selected kidney from half of the cats in each health group 2 times. Both observers were blinded to the identity, clinical information and health status of cats and renal images were measured in a randomized order using commercially available software (ie, OsiriX DICOM Viewer).

Renal length was measured from the cranial to the caudal pole of the kidney in the longitudinal plane with the kidney exhibiting an oval appearance and hyperechoic parallel bars formed by cross-sectioned pelvic diverticula. Corticomedullary thickness was evaluated in the transverse plane (where the characteristic “C”-sign of the renal crest is visible and the kidney appears rounded) and the dorsal plane. Cortical thickness was measured from the renal margin (excluding the renal capsule) to the outer border of the arcuate blood vessels (excluding the vessel); whereas RMT was measured from the arcuate blood vessels (including the vessel) to the hyperechoic renal crest (Figure A1A-C). The CM ratio was calculated in the transverse and dorsal plane by dividing the RCT by the RMT. In addition, renal shape, corticomedullary echogenicity, and renal pelvis were evaluated, and kidneys were visually checked for the presence of cavitary lesions, solid nodules or masses, dystrophic mineralization, segmental cortical lesions, medullary rim and band signs, and medullary striations.

### Statistical analysis

All statistical analyses were performed using SAS version 6.4 (SAS Institute Inc, Cary, NC, USA). Because analyses of left and right kidney yielded consistent results without evidence of laterality effects, renal dimensions were expressed as the mean of both kidneys for all analyses, except those evaluating kidney laterality and observer variability. Normality of the data distribution was tested using the Shapiro–Wilk test. A *P*-value < .05 was considered statistically significant. For post-hoc pairwise comparisons, *P-*values were adjusted using Tukey’s method to control for type I error.

A linear fixed effects model was used to compare renal dimensions across the disease groups. Transverse RCT was defined a priori as the primary endpoint based on earlier research.^2^ Disease group comparisons for this endpoint were based on predefined, biologically relevant pairwise contrasts between CKD, overall healthy, senior healthy and hyperthyroid cats. Results were reported as estimated mean differences and corresponding standard errors (as a measure of precision). Remaining renal dimensions were analyzed using the same fixed effects model as secondary, exploratory endpoints. Within the CKD group, differences in renal dimensions between IRIS stages were assessed using IRIS stage as the fixed factor. For the CKD group, scatter plots were generated to evaluate potential linear trends between renal function and renal dimensions.

Receiver operating characteristic (ROC) curve analysis was performed to evaluate the diagnostic accuracy of renal dimensions in differentiating the CKD group from the senior healthy group. Sensitivity was plotted against 1 − specificity. The area under the ROC curves (AUC) with the corresponding 95% CIs were calculated. Optimal cut-off values were assessed using the Youden index, which maximizes the sum of sensitivity and specificity.

A linear mixed model with kidney side as fixed effect and cat as random effect was fitted to determine the effect of kidney laterality on renal dimensions. The same analysis was repeated within each study group to assess within-group kidney side differences.

Multiple linear regression was used to assess the effect of age, and body weight (continuous variables) and sex (categorical variable) on renal measurements in the healthy group. Results were reported as estimated regression coefficient (β) ± SE. Post-hoc pairwise comparisons were conducted between sex categories.

The inter- and intraobserver agreement in measured renal dimensions was evaluated by fitting a random effects model with kidney, observer, and kidney-observer interaction as random effects. The interobserver intraclass correlation coefficient (ICC) was estimated by $\frac{{\hat{\sigma}}_k^2}{{\hat{\sigma}}_k^2+{\hat{\sigma}}_r^2+{\hat{\sigma}}_{kr}^2+{\hat{\sigma}}_e^2}$; and the intraobserver ICC by $\frac{{\hat{\sigma}}_k^2+{\hat{\sigma}}_r^2+{\hat{\sigma}}_{kr}^2}{{\hat{\sigma}}_k^2+{\hat{\sigma}}_r^2+{\hat{\sigma}}_{kr}^2+{\hat{\sigma}}_e^2}$, where ${\hat{\sigma}}_k^2$, ${\hat{\sigma}}_r^2$, ${\hat{\sigma}}_{rk}^2$, and ${\hat{\sigma}}_e^2$ are the restricted maximum likelihood estimates for the variance component due to the kidney, the observer, the kidney-observer interaction and the random error. Bland–Altman (BA) plots were used to assess inter- and intraobserver measurement differences, using the paired measurements of the 2 observers, and the 2 paired measurements of the second observer, respectively.

## Abbreviations

$\overline{d}$: mean difference
CM: corticomedullary
AUC: area under the curve
BA: Bland–Altman
CKD: chronic kidney disease
ECVDI: European College of Veterinary Diagnostic Imaging
ICC: intraclass correlation coefficient
IRIS: International Renal Interest Society
LoA: limits of agreement
RCT: renal cortical thickness
RI: reference interval
RMT: renal medullary thickness
ROC: receiver operating characteristic
sCr: serum creatinine
SDMA: symmetric dimethyl arginine
TT4: total thyroxine
USG: urine specific gravity
UPC: urine protein creatinine
