# Supplementary material for: Ultrasound-measured renal length and corticomedullary thickness for the diagnosis and staging of chronic kidney disease in cats
Source: J Vet Intern Med. 2026 Nov 10;40(6):aalag274. doi: 10.1093/jvimsj/aalag274 (PMC13750376; doi:10.1093/jvimsj/aalag274)
Supplement: Supplementary file 1 [file supplementary_material_aalag274.zip › Supplementary_material_aalag274/subtab1.docx]

|  | | **Junior**  **(1 – 2 years)** | **Young adult**  **(3 – 6 years)** | **Mature adult**  **(7 – 10 years)** | **Senior**  **(> 11 years)** | **CKD** | **Hyperthyroid** |
| --- | --- | --- | --- | --- | --- | --- | --- |
|  | | n = 45 | n = 45 | n = 43 | n = 46 | n = 38 | n = 42 |
| **Longitudinal image** | **Both** | 45 | 45 | 41 | 43 | 36 | 41 |
|  | **One** |  |  | 2 | 3 | 1 | 1 |
|  | **N/A** |  |  |  |  | 1 |  |
| **Dorsal image** | **Both** | 45 | 43 | 39 | 40 | 31 | 35 |
|  | **One** |  | 2 | 4 | 6 | 7 | 7 |
|  | **N/A** |  |  |  |  |  |  |
| **Transverse**  **image** | **Both** | 34 | 32 | 34 | 38 | 33 | 37 |
|  | **One** | 8 | 7 | 6 | 7 | 5 | 3 |
|  | **N/A** | 3 | 6 | 3 | 1 |  | 2 |

**Supplementary Table 1**: availability of ultrasonographic images of sufficient resolution to evaluate the renal dimensions (i.e., renal length in the longitudinal image, and cortical and medullary thickness in both dorsal and transverse image) in included cats classified by health (and additional age for the healthy cats).

Abbreviations: N/A, not available
