# Supplementary material for: Ultrasound-measured renal length and corticomedullary thickness for the diagnosis and staging of chronic kidney disease in cats
Source: J Vet Intern Med. 2026 Nov 10;40(6):aalag274. doi: 10.1093/jvimsj/aalag274 (PMC13750376; doi:10.1093/jvimsj/aalag274)
Supplement: Supplementary file 1 [file supplementary_material_aalag274.zip › Supplementary_material_aalag274/suptab2.docx]

**Supplementary Table 2**: prevalence of ultrasonographic renal abnormalities observed in the study sample, which is stratified by health status (and further by age for healthy cats). Reported abnormalities include irregular renal shape, cavitary lesions, solid nodules and masses, dystrophic mineralization, lithiasis, segmental cortical lesions, medullary rim- or band sign, reduced corticomedullary definition, increased cortical and medullary echogenicity, medullary striations, and pyelectasia (renal pelvic dilation > 2 mm). Prevalence is reported as absolute numbers; with percentages indicated in parentheses.

|  | **Junior**  **(1 – 2 years)** | **Adult**  **(3 – 6 years)** | **Mature adult**  **(7 – 10 years)** | **Senior**  **(> 11 years)** | **CKD** | **Hyperthyroid** |
| --- | --- | --- | --- | --- | --- | --- |
|  | n = 45 | n = 45 | n = 43 | n = 46 | n = 38 | n = 42 |
| **Irregular renal shape** | 0 | 0 | 2 (4.65%) | 3 (6.52%) | 20 (52.63%) | 1 (2.38%) |
| **Cavitary lesions** | 0 | 0 | 1 (2.33%) | 1 (2.17%) | 3 (7.89%) | 5 (11.90%) |
| **Solid nodules/masses** | 0 | 0 | 0 | 0 | 2 (5.26%) | 0 |
| **Dystrophic mineralisation** | 1 (2.22%) | 2 (4.44%) | 0 | 4 (8.70%) | 13 (34.21%) | 5 (11.90%) |
| **Lithiasis** | 0 | 2 (4.44%) | 3 (6.98%) | 4 (8.70%) | 17 (44.74%) | 8 (19.05%) |
| **Segmental Cortical Lesion** | 1 (2.22%) | 2 (4.44%) | 2 (4.65%) | 4 (8.70%) | 9 (23.68%) | 4 (9.52%) |
| **Medullary rim sign** | 21 (46.67%) | 19 (42.22%) | 13 (30.23%) | 13 (28.26%) | 6 (15.79%) | 14 (33.33%) |
| **Medullary band sign** | 2 (4.44%) | 4 (8.88%) | 2 (4.65%) | 7 (15.22%) | 7 (18.42%) | 8 (19.05%) |
| **Reduced corticomedullary definition** | 1 (2.22%) | 3 (6.66%) | 2 (4.65%) | 5 (10.87%) | 26 (68.42%) | 13 (30.95%) |
| **Increased cortical echogenicity** | 1 (2.22%) | 8 (17.77%) | 10 (23.26%) | 9 (19.57%) | 23 (60.53%) | 7 (16.66%) |
| **Increased medullary echogenicity** | 1 (2.22%) | 2 (4.44%) | 1 (2.33%) | 5 (10.87%) | 20 (52.63%) | 10 (23.81%) |
| **Medullary striations** | 1 (2.22%) | 4 (8.88%) | 4 (9.30%) | 4 (8.70%) | 11 (28.95%) | 11 (26.19%) |
| **Pyelectasia** | 0 | 0 | 0 | 0 | 5 (13.16%) | 0 |
