# Supplementary material for: Ultrasound-measured renal length and corticomedullary thickness for the diagnosis and staging of chronic kidney disease in cats
Source: J Vet Intern Med. 2026 Nov 10;40(6):aalag274. doi: 10.1093/jvimsj/aalag274 (PMC13750376; doi:10.1093/jvimsj/aalag274)
Supplement: Supplementary file 1 [file supplementary_material_aalag274.zip › Supplementary_material_aalag274/suptab3.docx]

**Supplementary Table 3:** estimated group-means ± standard deviations of left and right kidney dimensions separately (i.e., renal length, cortical and medullary thickness in dorsal and transverse plane and derived corticomedullary ratios in the dorsal and transverse plane) are presented for the overall sample and subsequently for each health group. Values are expressed in cm.

Within each group and for each kidney dimension, distinct superscripts indicate a statistically significant difference between left and right kidney (*P* < .05); whereas identical superscripts represent no significant difference between the left and right kidney.

|  | | **Total** | **Healthy** | **CKD** | **Hyperthyroid** |
| --- | --- | --- | --- | --- | --- |
|  | | n = 259 | n = 179 | n = 38 | n = 42 |
| **Renal length*** | **Left** | 3.75 ± 0.50^a^ | 3.69 ± 0.41^a^ | 3.09 ± 0.53^a^ | 3.82 ± 0.49^a^ |
|  | **Right** | 3.85 ± 0.50^b^ | 3.78 ± 0.41^a^ | 3.29 ± 0.50^a^ | 3.88 ± 0.45^a^ |
| **RCT dorsal**** | **Left** | 0.70 ± 0.15^a^ | 0.63 ± 0.13^a^ | 0.56 ± 0.15^a^ | 0.67 ± 0.15^a^ |
|  | **Right** | 0.68 ± 0.15^a^ | 0.62 ± 0.12^a^ | 0.51 ± 0.14^a^ | 0.67 ± 0.13^a^ |
| **RMT dorsal**** | **Left** | 1.15 ± 0.26^a^ | 1.08 ± 0.25^a^ | 0.82 ± 0.22^a^ | 1.07 ± 0.23^a^ |
|  | **Right** | 1.04 ± 0.26^b^ | 0.95 ± 0.20^b^ | 0.79 ± 0.19^a^ | 1.02 ± 0.22^a^ |
| **CM dorsal**** | **Left** | 0.63 ± 0.18^a^ | 0.61 ± 0.17^a^ | 0.71 ± 0.16^a^ | 0.66 ± 0.20^a^ |
|  | **Right** | 0.67 ± 0.18^b^ | 0.67 ± 0.17^b^ | 0.67 ± 0.19^a^ | 0.69 ± 0.21^a^ |
| **RCT transverse***** | **Left** | 0.65 ± 0.14^a^ | 0.63 ± 0.12^a^ | 0.56 ± 0.17^a^ | 0.67 ± 0.14^a^ |
|  | **Right** | 0.69 ± 0.14^b^ | 0.66 ± 0.13^b^ | 0.61 ± 0.14^a^ | 0.69 ± 0.13^a^ |
| **RMT transverse***** | **Left** | 1.18 ± 0.25^a^ | 1.21 ± 0.23^a^ | 0.99 ± 0.22^a^ | 1.22 ± 0.26^a^ |
|  | **Right** | 1.13 ± 0.25^b^ | 1.15 ± 0.24^b^ | 0.98 ± 0.26^a^ | 1.16 ± 0.23^a^ |
| **CM ratio transverse***** | **Left** | 0.59 ± 0.16^a^ | 0.54 ± 0.16^a^ | 0.57 ± 0.16^a^ | 0.57 ± 0.17^a^ |
|  | **Right** | 0.65 ± 0.16^b^ | 0.59 ± 0.15^b^ | 0.65 ± 0.17^b^ | 0.62 ± 0.18^a^ |

Abbreviations: CKD, chronic kidney disease; CM ratio, corticomedullary ratio; RCT, Renal cortical thickness; RMT, Renal medullary thickness.

*	based on n = 174 in the healthy group, n = 36 in the CKD group, and n = 41 in the hyperthyroid group

**	based on n = 167 in the healthy group, n = 31 in the CKD group, and n = 35 in the hyperthyroid group

*** based on n = 138 in the healthy group, n = 33 in the CKD group, and n = 37 in the hyperthyroid group
