# Supplementary material for: Ultrasound-measured renal length and corticomedullary thickness for the diagnosis and staging of chronic kidney disease in cats
Source: J Vet Intern Med. 2026 Nov 10;40(6):aalag274. doi: 10.1093/jvimsj/aalag274 (PMC13750376; doi:10.1093/jvimsj/aalag274)
Supplement: Supplementary file 1 [file supplementary_material_aalag274.zip › Supplementary_material_aalag274/suptab4.docx]

**Supplementary Table 4:** estimated group means ± corresponding standard deviations for renal dimensions (i.e., renal length, cortical and medullary thickness in the dorsal and transverse plane and corticomedullary ratios) in healthy cats classified by sex. Estimated group-level means are calculated from the mean measurements of left and right kidney of each cat (when available), as measured by the expert. They are expressed in cm.

Sexes with distinct superscripts differed significantly (*P* < .05). Sexes sharing one or two letters in the superscript did not significantly differ.

|  | **Healthy cats n = 179** | | | |
| --- | --- | --- | --- | --- |
|  | **M** | **MC** | **F** | **FC** |
|  | **n = 7** | **n = 60** | **n = 26** | **n = 86** |
| **Renal length** | 4.07 ± 0.38^a^ | 3.97 ± 0.33^a^ | 3.63 ± 0.37^b^ | 3.58 ± 0.31^b^ |
| **RCT Dorsal** | 0.69 ± 0.06^ab^ | 0.66 ± 0.13^a^ | 0.61 ± 0.10^ab^ | 0.60 ± 0.11^b^ |
| **RMT Dorsal** | 1.03 ± 0.16^ab^ | 1.05 ± 0.24^a^ | 0.93 ± 0.13^b^ | 1.03 ± 0.21^ab^ |
| **CM Dorsal** | 0.70 ± 0.14^a^ | 0.66 ± 0.17^a^ | 0.67 ± 0.12^a^ | 0.61 ± 0.13^a^ |
| **RCT Transverse*** | 0.68 ± 0.15^ab^ | 0.68 ± 0.13^a^ | 0.62 ± 0.09^ab^ | 0.62 ± 0.08^b^ |
| **RMT Transverse*** | 1.24 ± 0.24^ab^ | 1.24 ± 0.23^a^ | 1.04 ± 0.13^b^ | 1.17 ± 0.20^a^ |
| **CM Transverse*** | 0.58 ± 0.19^a^ | 0.58 ± 0.17^a^ | 0.61 ± 0.13^a^ | 0.55 ± 0.11^a^ |

Abbreviations: CM ratio, corticomedullary ratio; F, female; FC, female castrated; M, Male; MC, male castrated; RCT, Renal cortical thickness; RMT, Renal medullary thickness.

***** Based on n = 6 male, n = 57 male castrated, n = 23 female, n = 80 female castrated
